# A deep learning approach identifies new ECG features in congenital long QT syndrome

**DOI:** 10.1186/s12916-022-02350-z

**Published:** 2022-05-03

**Authors:** Simona Aufiero, Hidde Bleijendaal, Tomas Robyns, Bert Vandenberk, Christian Krijger, Connie Bezzina, Aeilko H. Zwinderman, Arthur A. M. Wilde, Yigal M. Pinto

**Affiliations:** 1 Department of Experimental Cardiology, Amsterdam UMC, Amsterdam, The Netherlands; 2 Department of Clinical Epidemiology Biostatistics and Bioinformatics, Amsterdam UMC, Amsterdam, The Netherlands; 3 Department of Cardiovascular Diseases, University Hospitals Leuven, Leuven, Belgium

**Keywords:** Deep learning, ECG, LQTS, Explainable AI

## Abstract

**Background:**

Congenital long QT syndrome (LQTS) is a rare heart disease caused by various underlying mutations. Most general cardiologists do not routinely see patients with congenital LQTS and may not always recognize the accompanying ECG features. In addition, a proportion of disease carriers do not display obvious abnormalities on their ECG. Combined, this can cause underdiagnosing of this potentially life-threatening disease.

**Methods:**

This study presents 1D convolutional neural network models trained to identify genotype positive LQTS patients from electrocardiogram as input. The deep learning (DL) models were trained with a large 10-s 12-lead ECGs dataset provided by Amsterdam UMC and externally validated with a dataset provided by University Hospital Leuven. The Amsterdam dataset included ECGs from 10000 controls, 172 LQTS1, 214 LQTS2, and 72 LQTS3 patients. The Leuven dataset included ECGs from 2200 controls, 32 LQTS1, and 80 LQTS2 patients. The performance of the DL models was compared with conventional QTc measurement and with that of an international expert in congenital LQTS (A.A.M.W). Lastly, an explainable artificial intelligence (AI) technique was used to better understand the prediction models.

**Results:**

Overall, the best performing DL models, across 5-fold cross-validation, achieved on average a sensitivity of 84 ± 2%, 90 ± 2% and 87 ± 6%, specificity of 96 ± 2%, 95 ± 1%, and 92 ± 4%, and AUC of 0.90 ± 0.01, 0.92 ± 0.02, and 0.89 ± 0.03, for LQTS 1, 2, and 3 respectively. The DL models were also shown to perform better than conventional QTc measurements in detecting LQTS patients. Furthermore, the performances held up when the DL models were validated on a novel external cohort and outperformed the expert cardiologist in terms of specificity, while in terms of sensitivity, the DL models and the expert cardiologist in LQTS performed the same. Finally, the explainable AI technique identified the onset of the QRS complex as the most informative region to classify LQTS from non-LQTS patients, a feature previously not associated with this disease.

**Conclusions:**

This study suggests that DL models can potentially be used to aid cardiologists in diagnosing LQTS. Furthermore, explainable DL models can be used to possibly identify new features for LQTS on the ECG, thus increasing our understanding of this syndrome.

**Supplementary Information:**

The online version contains supplementary material available at 10.1186/s12916-022-02350-z.

## Background

Congenital long QT syndrome (LQTS) is an inherited heart rhythm disorder characterized by a prolonged QT interval and abnormal T wave morphology on the electrocardiogram (ECG). QT prolongation predisposes those affected to life-threatening arrhythmias. These arrhythmias can lead to sudden loss of consciousness (syncope) and cardiac arrest and potentially cause sudden cardiac death. Although genetic heterogeneity is observed in LQTS, KCNQ1 (LQTS1) and KCNH2 (LQTS2), both encoding potassium channel proteins, and SCN5A (LQTS3), encoding a sodium channel protein, are the most common genes responsible for LQTS [1, 2].

LQTS is rare, with an estimated prevalence of 1:2000 among whites [3]. The diagnosis of LQTS is mainly based on the measurement of the QT interval corrected for heart rate (QTc) [1, 4]. Prolongation of the QT interval is the hallmark of LQTS, but it is not always present in carriers of a disease-causing mutation. In fact, many studies have shown that a substantial number of genotype positive LQTS patients have a baseline QT interval within normal limits (concealed LQTS). This makes it difficult, in particular, for a general cardiologist to diagnose LQTS, causing significant underdiagnosis of this disease [5–7]. Given that the degree of QT prolongation is associated with increased arrhythmic risk, concealed LQTS patients are still at risk of developing possibly lethal cardiac arrhythmias [5, 6]. Furthermore, specific medication can induce QT prolongation in these patients, increasing their risk of developing malignant arrhythmia and should therefore be avoided [2]. Over the years, the identification of additional ECG features, such as abnormal T wave morphology and the use of provocative testing strategies, helped unmask concealed LQTS patients [8–10]. However, this increased physician and public awareness of warning signs suggestive of LQTS also led to overdiagnosis of this disease. It has been shown that erroneous QTc calculation, mainly by non-arrhythmia specialists, is one of the primary reasons for individuals being misdiagnosed as having LQTS. Miscalculation of the QTc, misinterpretation of the normal distribution of QTc values, and misinterpretation of symptoms appear responsible for most of the diagnostic miscues [11]. Therefore, a more sensitive and reliable tool, able to aid cardiologists in diagnosing congenital LQTS on the ECG, could help improve the detection and early of this disease, making early intervention possible, hopefully preventing sudden cardiac death, for example, by reducing exposure to QT prolonging medication.

Previous studies using machine learning (ML) and deep learning (DL) methods have shown to improve the diagnosis of LQTS [12, 13]. These earlier studies presented high performing DL models; however, most studies were validated on the same population on which the DL models were trained, so the lack of external validation possibly limits the generalizability of the models in separate populations [13].

In this study, we evaluated if a DL algorithm can detect carriers of disease-causing mutations in LQTS. Instead of using the assessment of a LQTS expert, the genotype was used as the gold standard to train our DL models. To further assess the strength of our DL models, we validated the performance of the latter twice, using 5-fold cross-validation and with an external dataset. We then compared the performance of the DL models with conventional QTc measurements and with an expert cardiologist. Furthermore, we aimed to open the ‘black box’ of our DL models by using an explainable AI technique which can help us get more insight into what part of the ECG is most informative for the DL models.

## Methods

**Fig. 1:**
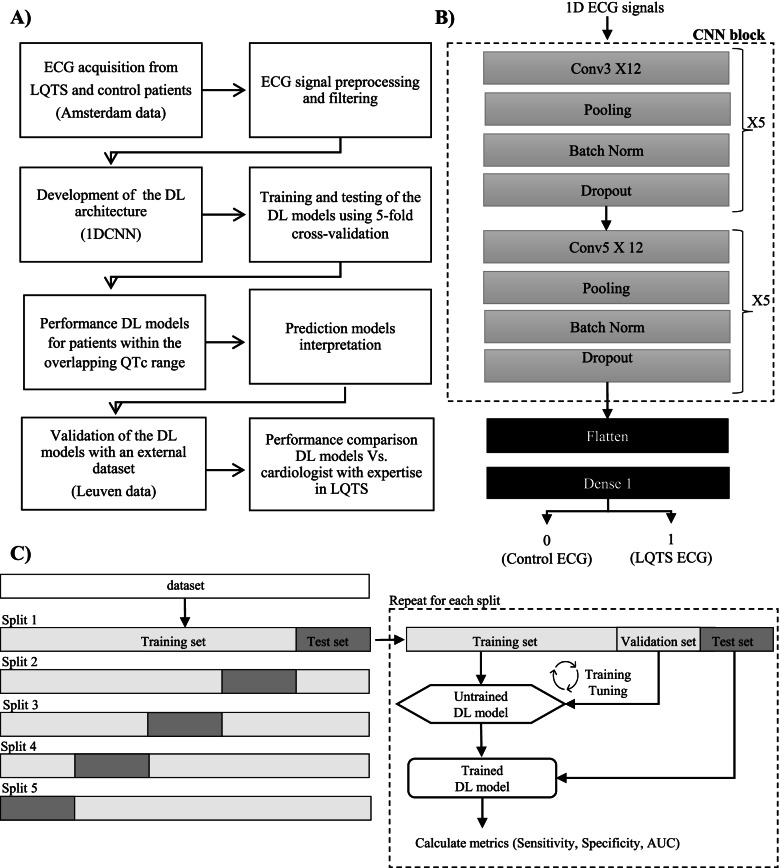
Study design for LQTS ECG classification. **A** Schematic representation of the implemented pipeline. **B** Proposed 1DCNN architecture. **C** Strategy used to train, validate, and test the DL models

Figure 1A summarizes the strategy we implemented to carry out this study.

Briefly, ECGs were collected, preprocessed, filtered, and used to train the DL models. In total, for each LQTS type, three separate training approaches were used, with each approach using a different type of input data. Figure 1B shows the developed 1DCNN architecture. For a more detailed version of the latter, see Additional file 1: Fig. S1. A binary classification was carried out for each class of LQTS type (i.e., LQTS1 = 1 versus control = 0, LQTS2 = 1 versus control = 0, LQTS3 = 1 versus control = 0). A 5-fold cross-validation procedure was used to train, validate, and test the performance of the DL models on the Amsterdam data (Fig. 1C). Subsequently, the performance of the DL models was evaluated only for LQTS and control patients whose QTc is within the overlapping QTc region. Furthermore, an explainable AI technique was used to better understand the prediction models. Finally, the models were validated on an external dataset (Leuven data), and the performances were compared with those of a cardiologist with expertise in LQTS (Fig. 1A).

A more detailed description of each phase is reported below.

### ECG acquisition—Amsterdam data

We collected ECGs from patients who were stored in the ECG database (MUSE v8, GE Healthcare) of the Amsterdam University Medical Centers (UMC), location Academic Medical Center, during the period from 1998 up to and including 2018, consisting of almost 1.5 million ECGs from almost 300,000 unique patients.

### ECG signal preprocessing and filtering

From this database, we extracted all ECGs from patients (age ≥ 16 years old) known to have a genetic variant with a class 4 or 5 classifications (according to ACMG guidelines) [14] in the *KCNQ1* gene (LQTS1), in the *KCNH2* gene (LQTS2) or in the *SCN5A* gene (LQTS3). These data were obtained from the LQTS database generated by Lahrouchi et al. [15] and the clinical genetics database of the Amsterdam UMC. ECGs were excluded if they were made on the emergency ward or during hospitalization on a clinical ward due to the fact that acute cardiac pathology could influence the ECG. ECGs from a total of 172 LQTS1, 214 LQTS2, and 72 LQTS3 patients were collected.

As a control group, instead of using ECGs of genotype negative controls, which would be of limited number, we decided to feed the DL models with more data. First, we selected from the MUSE database ECGs from patients who underwent general, non-cardiovascular pre-operative screening at the outpatient clinic. Then, we randomly selected 10000 ECGs labeled as ‘normal’ by the ECG machine's algorithm [16] to filter out ECGs with major abnormalities caused by other diseases and ECGs with artifacts acquired during the ECG signal acquisition. See Additional file 1: Methods for more details [16–19]. Given the estimated prevalence of LQTS syndrome being 1:2000 [3], we estimated that of the 10000 controls whose ECGs were used in this study, 9995 of them (99%) would be negative for LQTS mutations. The collected patient ECGs were then base64 decoded and resampled at the same sample frequency (i.e., 250 Hz). Furthermore, a QTc as calculated by the ECG machine's algorithm [16] (automatically measured QTc) was obtained for each ECG and used for further analysis.

### Development of DL architecture

We implemented a 1-dimensional convolutional neural network (1DCNN) architecture comprising 5 Conv1D layers, 5 Conv1D layers. Each Conv1D layer is followed by a batch normalization layer [20] to adjust and scale the input, MaxPooling1D layers, and a dropout layer [21] to prevent overfitting during the learning phase. There is a flatten and 1 dense layer. The classification training is carried out using binary-cross-entropy loss function and ADAM optimizer. For more details about the DL architecture, see Additional file 1: Fig. S1.

### Training and validation of the models

We trained the models using three approaches: (i) the First ECG approach, which uses the first acquired 12 lead ECG of each patient (i.e., a total of 172, 214 and 72 ECGs were used for LQTS 1, 2 and 3 respectively), (ii) the All ECG approach, which uses all acquired 12 leads ECGs of each patient (i.e., a total of 748, 1122 and 636 ECGs were used for LQTS 1, 2, and 3 respectively, for more details see Additional file 1: Fig. S2), and (iii) an additional approach called the Single Lead First ECG approach, for which a model is trained for each separate ECG lead, instead of using all the 12 leads together. For all the approaches, the DL model architecture stays constant, only the input data changes.

The models required a three-dimensional input formatted as follows: [*patient, time steps, features*]. In detail, for each patient, we used 12 leads (12 features) or a single lead at the time (1 feature) where each lead had 2500 amplitude values (timesteps). To train and test the DL models, we used a 5-fold cross-validation technique. In detail, the dataset was split into 5 folds of equal size, and each fold (which contained 20% of the ECGs of the initial dataset) was used as a test set once, while the other folds were temporarily combined to form a training set. The training set was further split into a training (80%) and validation set (20%) for hyperparameter optimization. Performance metrics on the test set were then calculated and stored. The process was repeated for the number of folds that have been generated. In each iteration, a new model was trained and tested. At the end of the 5-fold cross-validation run, the collected metrics of the 5 generated DL models were summarized with the mean and the standard deviation. The following metrics were collected: sensitivity, specificity, and area under the curve receiver operator characteristic (AUC-ROC). We deemed the best performing model the one with the highest AUC of the 5-fold cross-validation run. To address the class imbalance, we oversampled the minority class (LQTS) so that the same proportions of control and LQTS patients were present in the training set. The test set was not modified and remained unbalanced. To note that a patient is identifiable by a unique ID number and only the first or all ECGs available of a patient would appear exclusively in the training or testing set. We trained the models for 50 epochs, using a batch size of 32. A value of 0.5 was chosen as the best threshold for ECG classification. This implied that ECGs with probability scores above 0.5 were classified as LQTS, while ECGs with probability scores below 0.5 were classified as controls.

### Prediction model interpretation

To better understand the prediction models, we used the guided Grad-CAM [22] technique on 1DCNN model to build a ponderated activation map according to gradient’s importance. If an activation map has a large gradient, it means the activated region has a large impact on the decision. In detail, to build a ponderated map, we retrieved the gradients with respect to the activation maps from the first convolutional layer of our models. The best performing DL models for LQTS 1, 2, and 3 of the 5-fold cross-validation and trained using 12-lead ECGs (first acquired or all ECGs) were used. The activation maps were built considering all leads in the ECGs. We then set to zero the gradients associated with a negative value of the activation maps, calculated the average gradient (weight) associated with each filter, and then multiplied each filter by its corresponding weight and summed up all the filters to get the final map. The values of ponderated activation map were then normalized between 0 and 1. To retrieve the values from the ponderated map corresponding to the different ECG regions (P waves, QRS complexes, and the S segments with the T waves), we used lead I as a reference. In detail, we overlaid lead I with the ponderated map and retrieved the corresponding values (see next paragraph to see how to identify the different ECG regions). Finally, from each range, we took the max values and calculated the average of the max values (called score Grad-CAM from now on) for each wave type.

### Identification of ECG region (P wave, QRS complex, S segment, and T wave)

We used the heartbeat detection algorithm implemented in Python py-ecg-detectors 1.0.2 [23] to identify the R peaks in each ECG recording. Before the detector could be used, the class had to be initialized with the sampling rate of the ECG recording. We initialized it to 250 hertz and used the Engelse and Zeelenberg algorithm to detect the R peaks using lead I as a reference. The output of the detector is an array with the index of the corresponding R peaks. We then retrieved the P wave from the R peak with the following formula: index R peak − 50, index R peak − 11. This interval corresponds to 49 amplitude values, which are 0.16 s in our ECG recording. For the QRS complex, we used the following formula: index R peak − 10, index R peak + 15. This interval corresponds to 25 amplitude values which are 0.10 sec in our ECG recording. Finally, we used the following formula for the S segment and the T wave: index R peak + 16, index R peak + 95. This interval corresponds to 80 amplitude values, which are 0.32 sec in our ECG recording.

### QRS complex comparison

The QRS complexes from lead I were identified using the strategy mentioned in the above paragraph. In detail, the first 2 and the last 2 R peaks identified by the algorithm were excluded from the analysis keeping approximately 10 R peaks per patient. From these, we retrieved the corresponding 10 QRS complexes and calculated the median QRS complex per patient. Finally, the median QRS complex of control patients was compared to the median QRS complex of the LQTS patients.

### External validation set—Leuven data

The external validation dataset was collected from the University Hospital Leuven, Belgium. They were collected following the same criteria as presented for the Amsterdam data. The dataset included 32 genotype-positive LQTS1, 80 genotype-positive LQTS2, and 2280 ECGs control patients.

### Evaluation by an expert cardiologist

A subset of control ECGs (*n* = 150 X 2), LQTS1 (*n* = 30), and LQTS2 (*n* = 30) ECGs were randomly extracted from the Leuven data and were visually evaluated by a cardiologist with expertise in electrophysiology and LQTS (A.A.M.W.). The expert classified the ECGs in LQTS1/2 or non-LQTS. The expert did this by measuring the QTc and assessed if there were any morphological T-wave abnormalities present associated with either LQTS1 or 2. If the QTc was normal and in the absence of T-wave abnormalities, the ECG was classified as non-LQTS by the expert.

### Statistical analysis

We used a two-sided paired sample *t*-test to compare the scores Grad-CAM corresponding to different ECG regions. The *scipy.stats.ttest_rel()* function in python was used for this purpose. We then corrected the *p*-value for multiple testing. We used the Wilcoxon signed-rank test to compare the performances of the models.

## Results

### Data collection

The Amsterdam data included ECGs from a total of 172 LQTS1, 214 LQTS2, 71 LQTS3, and 10,000 control patients. For an overview of all baseline characteristics of the selected patients, see Additional file 1: Table S1. Overall, the LQTS group had, as expected, a significantly higher mean QTc compared to controls (452 ± 35 ms compared to 411 ± 17 ms, respectively; *p* < 0.0001). The proportion of cases with prolonged QTc is 51%, 39%, and 43% for LQTS1, LQTS2, and LQTS3, respectively. The proportion of controls with prolonged QTc is 0.01%. Thresholds used to define prolonged QTc were as follows: ≥ 450 ms for males, QTc ≥ 460 ms for females. Each ECG consisted of 10 sec for each of 12 leads. Data was collected with a sampling frequency of either 250 or 500hz, and all data was downsampled to 250 hz. Finally, for an overview of the different types of genetic variants for each LQTS type and an overview of the specific genetic variants prevalent in the Amsterdam data, see Additional file 1: Table S2-3.

### Performance of first ECG and all ECG models on Amsterdam data—internal validation

All three of the approaches (First ECG, All ECG, and Single Lead First ECG) were used to develop the DL models. Table 1 shows the average results of 5-fold cross-validation obtained for the First ECG models. Using this approach, the models achieved an average AUC of 0.88, 0.89, and 0.79 for LQTS 1, 2, and 3, respectively. Probability scores from which the predictions were derived are shown in Additional file 1: Fig. S3. For the All ECG approach, the DL models achieved an average AUC of 0.90, 0.92, and 0.89 for LQTS1, 2, and 3, respectively. Probability scores from which the predictions were derived are shown in Additional file 1: Fig. S4. When comparing the performance of the First ECG models to the All ECG models, in addition to performing better (in particular for LQTS3, AUC *p*-value < 0.05), the All ECG models also showed a decreased standard deviation (SD) of the performance statistics especially for sensitivity. This suggests that the models performed similarly on 5-fold cross-validation and were better able to generalize. No sex-specific differences in the DL model's performance were found.

**Table 1:** Model performances on Amsterdam data

Training	Type	Internal validation (Amsterdam data)		
		Sensitivity ± SD	Specificity ± SD	AUC ± SD
First ECG approach (Amsterdam data)	LQTS1	79 ± 9%	96 ± 1%	0.88 ± 0.04
	LQTS2	89 ± 7%	90 ± 3%	0.89 ± 0.03
	LQTS3	67 ± 18%	90 ± 9%	0.79 ± 0.05
All ECG approach (Amsterdam data)	LQTS1	84 ± 2%	96 ± 2%	0.90 ± 0.02
	LQTS2	90 ± 2%	95 ± 1%	0.92 ± 0.01
	LQTS3	87 ± 6%	92 ± 4%	0.89 ± 0.03

The mean of the collected metrics and the corresponding standard deviation (SD) of the 5-fold cross-validation is reported. First ECG approach: the DL models were trained using the first acquired 12-lead ECGs. All ECG approach: the DL models were trained using all acquired 12-lead ECGs (not only the first acquired) per patient

Furthermore, when comparing sensitivity of the First ECG models with that diagnosing LQTS using automatic QTc measurement (Additional file 1: Table S1), the DL models performed better: 51% versus 79% for LQTS 1, 39% versus 89% for LQTS 2, and 43% versus 67% for LQTS 3. This improvement was even greater in comparison to the All ECG models. To validate the accuracy of the automatically measured QTc, we performed the same analysis; however, this time, we manually measured the QTc (only for LQTS patients) and then identified the proportion of LQTS patients with prolonged QTc (Additional file 1: Table S1). The DL models again outperformed the QTc measurement in detecting LQTS patients. For more details about automatically and manually measured QTc, see Additional file 1: Methods.

Furthermore, to evaluate model confidence in patients with normal QTc compared to patients with prolonged QTc, probability scores were extracted for the First ECG models. Overall, the results showed that the DL models identified a high proportion of LQTS ECGs with high confidence in both groups. More in detail, an average of 87% of all LQTS ECGs with prolonged QTc and an average of 71% of all LQTS ECGs with normal QTc had a probability score ≥ 0.70.

### Performance of single Lead first ECG models on Amsterdam data

Aside from the two approaches using all 12 leads together, the Single Lead First ECG approach was also used to train and test the models. Interestingly, for LQTS 1, 2, and 3, the performance of all the models using a single lead approximated the performance when using the First ECG approach. For the performance of all these Single Lead First ECG models, see Additional file 1: Tables S4-6.

### Performance of DL models for patients within the overlapping QTc range

One of the problems in diagnosing LQTS relates to the overlap in the QTc values between LQTS mutation carriers and healthy controls. In our study cohort, we found approximately 40% overlap between the QTc value distribution of the two populations (Additional file 1: Fig. S5). The overlapping region was defined as the region between the lower limit of QTc values distribution of the LQTS patients ranging to the upper limit of the QTc value distribution of the controls. We wanted to evaluate how the First ECG and All ECG models performed in classifying patients within the overlapping QTc region. In detail, we restricted the analysis to only those control and LQTS patients whose QTc values were within the 10th–90th percentile of the overlapping QTc region. QTc ranges analyzed are 398-475 ms, 403–477 ms, and 403–475 ms for LQTS 1, 2, and 3, respectively. Predictions of the First ECG and All ECG models were then retrieved, and the corresponding performance metrics recalculated (Additional file 1: Table S7). Overall, no statistical differences were found compared to the performance of the DL models, which included all LQTS and control patients.

### Identification of ECG features importance

**Fig. 2:**
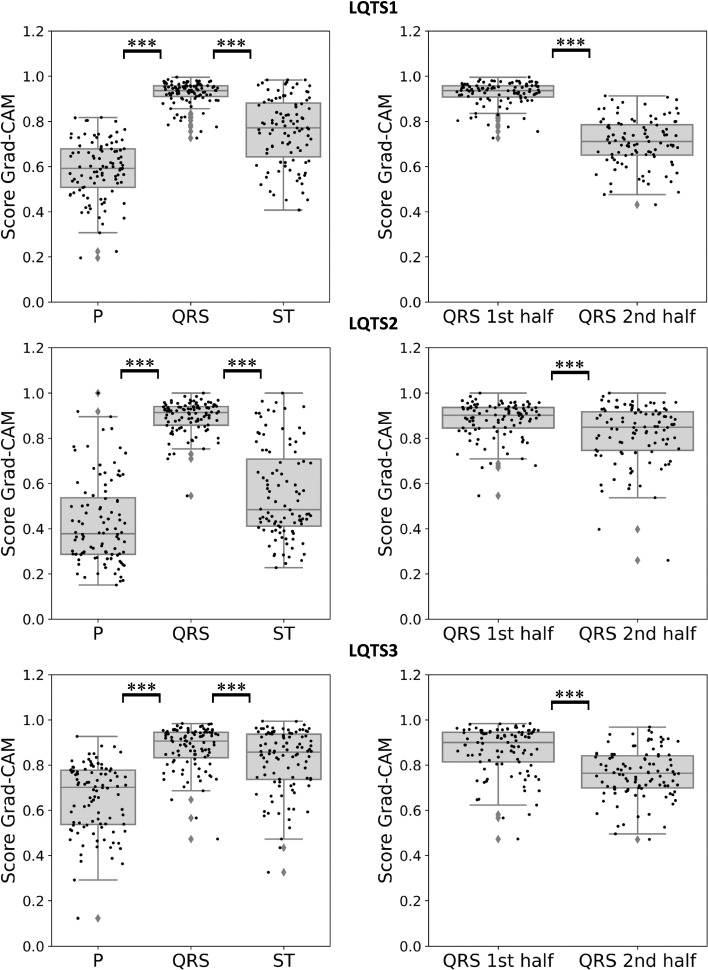
Identification of ECG features importance. Box plots showing the score Grad-CAM corresponding to the P wave, QRS complex, and the S segment with the T wave calculated for 100 control ECGs correctly classified by the best performing DL models developed for **A** LQTS1, **B** LQTS2, and **C** LQTS3 ECG classification. *** Adjusted p-values ≤.001

To investigate how informative each ECG region was, we used an explainable AI technique and retrieved a score Grad-CAM for each wave type (see Methods for more details). Only a subset of our study cohort was used for this analysis. In detail, 100 control patients of the testing fold correctly classified (probability score ≤ 0.05) by the best performing First ECG models of the 5-fold cross-validation were used. The results of this analysis showed that the most relevant region of the ECG used by the First ECG models to distinguish control and LQTS 1 or 2 patients corresponds to the QRS complex (adjusted *p*-values < 0.001). On the contrary, the score Grad-CAM of the T wave showed high variability, as well as of the P wave. For LQTS3, the QRS complex and the T wave seemed both relevant compared to P wave, with the QRS being the most relevant region (adjusted *p*-value < 0.001) (Fig. 2, left). A more detailed analysis of the QRS complex showed that the first half (i.e., onset) of the QRS complex was more relevant than the second half for ECG classification for LQTS 1, 2 and 3 (adjusted *p*-value < 0.001) (Fig. 2, right).

**Fig. 3:**
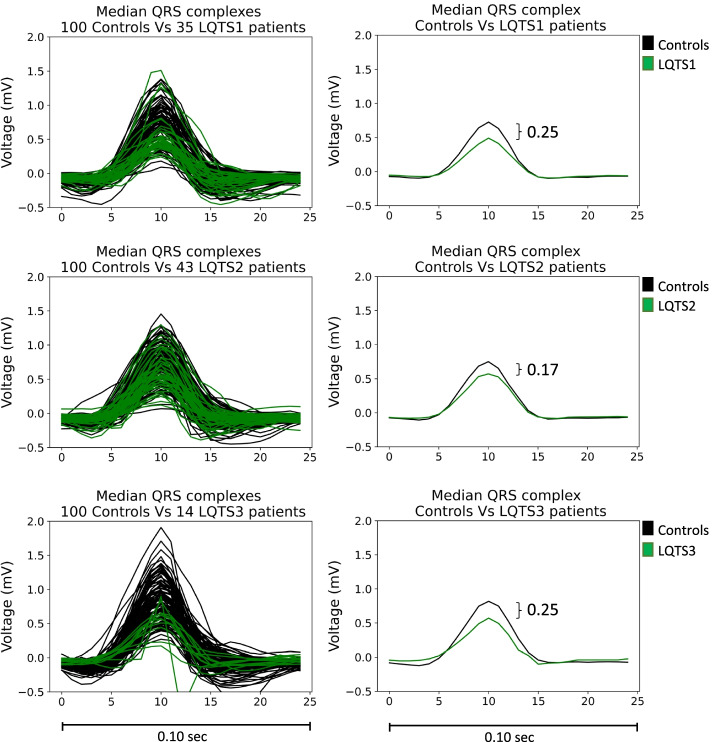
QRS complex comparison. The median QRS complex of 100 control ECGs (black lines) was retrieved and compared to the median QRS complex of the corresponding LQTS1/2/3 ECGs (green lines) analyzed by the best performing DL models (left). The median QRS complex from the control ECGs and LQTS ECGs was then calculated (right). On the *x*-axis data points from the waveform are shown; 25 data points correspond to 0.10 s or 100 ms

To better visualize the difference, we retrieved the QRS complexes from lead I from the same control patients analyzed above and the LQTS 1, 2 and 3 patients and calculated the median QRS complex for each patient (Fig. 3, left). The median QRS complex from all the control and all LQTS ECGs was then calculated (Fig. 3, right). We found a lower amplitude of the median QRS complex in all 3 types of LQTS patients compared to the control group (delta = 0.17-0-25 mV).

The same results were obtained when using the best performing DL models trained on all acquired 12-lead ECGs (All ECG approach) (data not shown).

### Performances of the model on Leuven data—external validation

The Leuven data included ECGs from 32 LQTS1, 80 LQTS2, and 2200 control patients. For an overview of all baseline characteristics, see Additional file 1: Table S8. We validated the First ECG and All ECG models on this dataset and found that the performances held up with this novel external cohort (Table 2). The First ECG models achieved an average AUC of 0.86 and 0.87 for LQTS 1 and 2, respectively. The All ECG models achieved an average AUC of 0.90 and 0.89 for LQTS type 1 and 2, respectively. A comparison of the DL model's performances showed a significant improvement for the models trained on all available 12-lead ECGs for LQTS1 (AUC *p*-value < 0.05).

**Table 2:** Model performances on Leuven data

Training	Type	External validation (Leuven data)		
		Sensitivity ± SD	Specificity ± SD	AUC ± SD
First ECG approach (Amsterdam data)	LQTS1	80 ± 2%	94 ± 2%	0.86 ± 0.01
	LQTS2	92 ± 3%	81 ± 6%	0.87 ± 0.02
All ECG approach (Amsterdam data)	LQTS1	87 ± 4%	93 ± 3%	0.90 ± 0.01
	LQTS2	90 ± 4%	88 ± 3%	0.89 ± 0.01

The whole set of LQTS1 (*n* = 32), LQTS2 (*n* = 80), and controls (*n* = 2280) from the Leuven dataset was used to validate our DL models, which were trained on the Amsterdam data using the only the first acquired 12-lead ECGs (i.e., First ECG approach) (top) or all acquired 12-lead ECGs (i.e., ALL ECG approach) (bottom). The mean and standard deviation (SD) of the collected metrics is reported

For an overview of the different types of genetic variants for each LQTS type and an overview of the specific genetic variants prevalent in the Leuven data, see Additional file 1: Table S9-10. The most prevalent variants and distribution of variant types are different in both Amsterdam (Additional file 1: Table S2-3) and Leuven datasets (Additional file 1: Table S9-10), meaning the models generalize well, also in a population with different LQTS associated genetic variants.

### Performance comparison—DL model versus. Cardiologist

A subset of 30 LQTS1, 30 LQTS2, and 300 control ECGs (i.e., 150 × 2), which were randomly selected from the Leuven dataset, were blindly assessed by the expert cardiologist. The same set was also analyzed by the First ECG and All ECG models. The results are presented in Table 3. Overall, our best performing models (trained on all acquired 12-lead ECGs) outperformed the cardiologist in terms of specificity. In terms of sensitivity, the cardiologist and the models performed the same.

**Table 3:** Performance comparison DL model versus cardiologist on LQTS 1 and 2

Training	Type	External validation (Leuven data)		
		Sensitivity ± SD	Specificity ± SD	AUC ± SD
All ECG approach (Amsterdam data)	LQTS1	89 ± 4%	97 ± 3%	0.93 ± 0.02
	LQTS2	91 ± 3%	87 ± 2%	0.89 ± 0.02
Expert cardiologist in LQTS	LQTS1	93%	90%	0.90
	LQTS2	90%	80%	0.85

A subset of 30 LQTS1, 30 LQTS2, and 300 controls (150 X 2) from the Leuven dataset was selected and used to validate our DL models, which were trained on the Amsterdam data using all acquired 12-lead ECGs (i.e., ALL ECG approach) per patient (top). The mean and standard deviation (SD) of the collected metrics is reported. The same subset was evaluated by an expert cardiologist in LQTS (bottom)

## Discussion

This study presents DL models trained to identify genotype positive LQTS patients from ECG. The DL models showed robust performances that achieved approximately an AUC of 0.90 and optimal generalization properties when tested on an unseen cohort. Furthermore, the DL models also showed to outperform in terms of sensitivity conventional QTc measurement, thus showing the potential to identify concealed LQTS patients. Interestingly, we found that the DL models are looking at additional ECG features, particularly at the onset of the QRS complex, rather than only at the QT interval, for ECG classification. Therefore, we potentially identified a new electrocardiographic region that can contribute to the improvement of LQTS diagnosis.

In detail, we developed 1DCNN binary classification models trained with a large 10-s 12-lead ECGs dataset provided by Amsterdam UMC composed of 10000 controls and 458 genetically proven LQTS patients. The use of ECGs of genetically proven LQTS is one of the major strengths of this study. Using the genotype as the gold standard, our models can possibly identify LQTS patients whose ECGs do not show any apparent phenotype as judged by a human expert and, therefore, in a normal circumstance, could remain undiagnosed. This approach allows models to outperform the expert, adding to the cardiologist’s evaluation rather than being trained by it.

Overall, on the Amsterdam data, in particular with the All ECG models, robust performances were obtained in predicting all three types of LQTS with small variability across 5-fold cross-validation, suggesting that the models had good generalizability. Our best models, trained on all acquired 12-lead ECGs of each patient, achieved an average sensitivity for the LQTS 1, 2, and 3 of 84%, 90%, and 87%, a specificity of 96%, 95%, and 92%, and an AUC of 0.90, 0.92 and 0.89 respectively. DL is a technique that performs best on voluminous data, and our results showed that by augmenting data and using more ECGs per patient (e.g., using all acquired 12-lead ECGs instead of only the first acquired 12-lead ECGs), we increased the training data for our models, which led to better results.

Further analysis using single-lead ECGs showed that the models built on single-lead information could also predict LQTS ECG with minimal reduction in performance compared to the First ECG models. This result suggests that every single lead can harbor key features and that can potentially be used for LQTS diagnosis. The performances of the DL models were also tested on an external data set (Leuven data) to evaluate how generalizable our models are to a separate patient population. The overall performances obtained on the Leuven data were comparable to the performances obtained on the Amsterdam data, confirming the ability of the developed DL model to detect genotype positive LQTS patients. Additionally, the current models have also shown to outperform an expert cardiologist in LQTS, in terms of specificity, while in terms of sensitivity the DL models and the expert cardiologist performed the same. In theory, this could mean that the outcome of DL models could mimic the evaluation of an international expert on the ECG diagnosis of LQTS and could be implemented in clinical care, for example, in the form of a clinical decision tool to aid general cardiologists in diagnosing LQTS on the ECG.

Besides the expert cardiologist, if we compare the performance of the DL models to earlier published approaches that aim to increase the performance of detecting genetic LQTS, the DL models reached almost similar diagnostic accuracy as the Brisk Standing Test (AUC 92%) [9], measuring QT-interval during exercise (AUC 0.93) [8] and individualized QT interval measurement with Holter (AUC 0.96) [24]. These methods, however, need additional testing compared to the DL models that only require a 12-lead resting ECG as input, thus making the latter possibly more time and cost-effective while reaching almost similar performance. Furthermore, these additional methods are usually not performed in cases with a normal QTc on the resting ECG or when they are mostly asymptomatic and may therefore remain undiagnosed. This would not be the case with our DL models because it only uses the resting ECG itself.

It is important to identify concealed LQTS mutation carriers because they might still be at risk for malignant arrhythmias under certain circumstances, such as the use of QT-prolonging drugs or in case they pass the mutation to their children, which can become severely symptomatic while remaining undiagnosed [25]. In this study, we showed that DL models could help identify LQTS mutation carriers with normal QTc. A comparison between DL model's performance and QTc measurements showed that the DL models outperformed the latter in predicting all three types of LQTS. In detail, the DL models identified a proportion of LQTS patients that were missed by the QTc measurement due to not passing the QTc threshold. Interestingly an average of 87% of LQTS patients with prolonged QTc and an average of 71% of patients with normal QTc were predicted with high confidence (probability score ≥ 0.70). Finally, we analyzed the DL model’s performance in classifying control and LQTS patients whose QTc values were within the 10th–90th percentile of the overlapping region of the QTc values distribution of the two populations and showed that the DL models were still able to distinguish controls from LQTS patients with an average AUC of 0.88 for the models trained on all acquired 12-lead ECGs.

Overall, these findings suggest that the models use additional ECG features rather than only the QT interval for LQTS ECG classification.

Model explainability is one of the challenges in machine learning, especially for deep learning models, where algorithms usually operate as black boxes, and it is unclear how a certain decision is derived. Explaining the DL model decision and which features are the most salient in a model's predictions is important to understand the value and accuracy of the findings. To the best of our knowledge, no study has attempted to explain the prediction models and understand the decision made by the DL model for LQTS ECG classification. Therefore, we applied an explainable AI technique, a guided Grad-CAM [22] approach adapted to work on our 1DCNN, and then calculated a score to localize the most important region used by the DL models for ECG classification. Surprisingly, with this method, we found that the QRS complex, particularly the onset of the QRS complex, is the most relevant region for ECG classification. LQTS is a condition that has been associated with defects of the repolarization phase of the action potential and T-wave abnormalities [26]. Even though the T wave morphology is a known and most often used feature for LQTS diagnosis, it does not always emerge in our models. An explanation might be that the model does not give so much weight to the T wave due to the large inter-individual variability of this part.

No studies as of yet have found low QRS amplitude or other (morphological) QRS features to be associated with LQTS 1, 2, or 3. Abnormalities at the onset of the QRS complex and the QRS complex, which are mainly associated with depolarization, are unexpected within the context of LQTS. The finding that our DL models derives much from the initial part of the QRS collides with the current concepts that translate how ion channel dysfunction leads to ECG abnormalities by changing individual action potentials. Our finding suggests that the initial myocardial depolarization of the tissue as a whole might be altered in carriers of a disease-causing LQTS mutation. To our current knowledge, mutations associated with LQTS1 and LQTS2 are thought to alter solely myocardial repolarization, and therefore, depolarization changes are hard to explain within the known pathophysiology of these LQTS types. However, low R-wave amplitude or QRS voltages are seen in both ischemic and genetic cardiomyopathy, with genetic mutations in the phospholamban protein being known to cause lowering of QRS voltages over time, which is thought to predispose myocardial fibrosis and dilation of the heart [27, 28]*.* However, to our best knowledge, this phenomenon is not known to occur in patients with LQTS and is difficult to understand. Other known causes for low QRS voltage are pericardial or pleural effusion, pulmonary emphysema, and obesity, among many others [29]. In the dataset we used as our control group, data regarding medical history, echocardiographic status, or body mass index were not available; therefore, it was not possible to evaluate if these factors could have influenced the QRS complex amplitude.

It is important to note that we could only look at the first convolutional layer of our DL models. Only the Grad-CAM scores from this first layer could be overlaid with the ECG and make the predictions interpretable. Deeper layers extract a combination of low-level features, which are finally used for LQTS classification. In our DL models, the presence of max pooling layers made it challenging to trace back the gradients from deeper layers to the ECG.

Furthermore, in this study, we only used ECGs that were labeled as normal by the ECG’s machine algorithm as a control set. Although this allowed us to reduce the complexity for model explainability and focus more on what the DL models see as abnormal on the LQTS ECGs, the selection of these controls might limit the generalizability of the DL models on the general population. Future work includes training and testing the DL models on an unselected population of patients, which also include pediatric ECGs. LQTS is often diagnosed before the age of 16 years, and our DL models were only trained and tested on adult ECGs. Finally, our models were trained and validated on a dataset consisting of patients from mostly European ethnicity and ECG made with an ECG machine for one manufacturer only. When implementing these models outside of our population, generalizability issues could occur and further external validation or retraining using data from other ethnicities and other ECG machines could be necessary.

## Conclusions

In conclusion, this study showed that DL can improve diagnosis of LQTS and potentially serve as a very effective screening tool to identify genotype positive LQTS patients (with normal and prolonged QTc) and therefore help a general cardiologist predict which patient might need further workup without having to consult an expert, thus improving LQTS diagnosis. Furthermore, we took the first steps to understand what our DL models learned and used for LQTS ECG classification, revealing a potentially new electrocardiographic feature for LQTS diagnosis.

Tools that can automatically detect LQTS harboring disease-causing variants and identify ECG features associated with this disease, as we present in this paper, could be of great clinical importance in aiding cardiologists in diagnosing LQTS and understanding the pathophysiology of inherited heart disease general.

## Supplementary Information

**Additional file 1: Table S1.** Demographics of the study cohort - Amsterdam data. **Table S2.** Distribution of different types of genetic variants for LQTS 1, 2 and 3 – Amsterdam data. **Table S3.** Prevalence of specific genetic variants for LQTS 1, 2 and 3 - Amsterdam data. **Table S4.** Single Lead First ECG model performances for LQTS1 - Amsterdam data. The mean of the collected metrics and the corresponding standard deviation (SD) of the 5-fold cross-validation is reported. The DL models were trained using single leads of the first acquired ECG of control and LQTS1 patients. **Table S5.** Single Lead First ECG model performances for LQTS2 - Amsterdam data. The mean of the collected metrics and the corresponding standard deviation (SD) of the 5-fold cross-validation is reported. The DL models were trained using single leads of the first acquired ECG of control and LQTS2 patients. **Table S6.** Single Lead First ECG model performances for LQTS3 - Amsterdam data. The mean of the collected metrics and the corresponding standard deviation (SD) of the 5-fold cross-validation is reported. The DL models were trained using single leads of the first acquired ECG of control and LQTS3 patients. **Table S7.** Model performances for LQTS 1, 2, and 3 - Amsterdam data. The performance of the DL models for patients whose QTc is within the 10^th^-90^th^ percentile of the overlapping QTc region is reported. The QTc ranges analyzed are 398-475 ms, 403-477 ms, and 403-475 ms for LQTS 1, 2, and 3, respectively. The automatically measured QTc was used (thresholds used to define prolonged QTc: ≥ 450 ms for males, QTc ≥ 460 ms for females). The mean of the collected metrics and the corresponding standard deviation (SD) of the 5-fold cross-validation is reported. First ECG approach: the DL models were trained using the first acquired 12-lead ECGs. All ECG approach: the DL models were trained using all acquired 12-lead ECGs (not only the first acquired) per patient. **Table S8.** Demographics of the study cohort (Leuven data). **Table S9.** Distribution of different types of genetic variants for LQTS 1 and 2 – Leuven data. **Table S10.** Prevalence of specific genetic variants for LQTS 1 and 2 - Leuven data. **Fig. S1.** 1DCNN architecture. Schematic representation of the proposed 1DCNN architecture. The model was trained using 12-lead ECGs or single lead. The number 12 in the second column represents the 12-lead ECGs, while the number 1 in the bracket represents the use of the single lead. All 12 leads individually were used. **Fig. S2.** ECG distribution. Bar plots showing the number of ECGs for LQTS 1, 2, and 3 patients used in the ALL ECG approach. **Fig. S3.** Probability score distribution of control and LQTS patients. Box plots showing the probability score distribution for A) LQTS1, B) LQTS2, and C) LQTS3 and control patients obtained from the DL models trained on the first acquired 12-lead ECGs. The red dotted line represents the threshold used for the ECG classification. **Fig. S4.** Probability score distribution of control and LQTS patients. Box plots showing the probability score distribution of A) LQTS1, B) LQTS2, and C) LQTS3 and control patients obtained from the DL models trained on all acquired 12-lead ECGs. The red dotted line represents the threshold used for the ECG classification. **Fig. S5.** QTc value distribution (Amsterdam data). Top: QTc value distributions of LQTS versus control patients. Bottom: table showing the mean, the lower, and the upper limit of the above QTc value distributions. The automatically measured QTc was used (thresholds used to define prolonged QTc: ≥ 450 ms for males, QTc ≥ 460 ms for females).

## Data Availability

Given its personally sensitive nature, the data used in this study is not publicly available. The code used to build the 1DCNN architecture and the best trained models are, however available at the following GitHub page: https://github.com/Aufiero/aiecglqts.

## Abbreviations

DL: Deep learning
ML: Machine learning
ECG: Electrocardiogram
1DCNN: 1D convolutional neural network
AUC: Area under the curve
Grad-CAM: Gradient class activation mapping
SD: Standard deviation
AI: Artificial intelligence
