# Supplementary material for: A deep learning approach identifies new ECG features in congenital long QT syndrome
Source: BMC Med. 2022 May 3;20:162. doi: 10.1186/s12916-022-02350-z (PMC9063181; doi:10.1186/s12916-022-02350-z)
Supplement: Supplementary file 1 — Additional file 1: Table S1. Demographics of the study cohort - Amsterdam data. Table S2. Distribution of different types of genetic variants for LQTS 1, 2 and 3 – Amsterdam data. Table S3. Prevalence of specific genetic variants for LQTS 1, 2 and 3 - Amsterdam data. Table S4. Single Lead First ECG model performances for LQTS1 - Amsterdam data. The mean of the collected metrics and the corresponding standard deviation (SD) of the 5-fold cross-validation is reported. The DL models were trained using single leads of the first acquired ECG of control and LQTS1 patients. Table S5. Single Lead First ECG model performances for LQTS2 - Amsterdam data. The mean of the collected metrics and the corresponding standard deviation (SD) of the 5-fold cross-validation is reported. The DL models were trained using single leads of the first acquired ECG of control and LQTS2 patients. Table S6. Single Lead First ECG model performances for LQTS3 - Amsterdam data. The mean of the collected metrics and the corresponding standard deviation (SD) of the 5-fold cross-validation is reported. The DL models were trained using single leads of the first acquired ECG of control and LQTS3 patients. Table S7. Model performances for LQTS 1, 2, and 3 - Amsterdam data. The performance of the DL models for patients whose QTc is within the 10th-90th percentile of the overlapping QTc region is reported. The QTc ranges analyzed are 398-475 ms, 403-477 ms, and 403-475 ms for LQTS 1, 2, and 3, respectively. The automatically measured QTc was used (thresholds used to define prolonged QTc: ≥ 450 ms for males, QTc ≥ 460 ms for females). The mean of the collected metrics and the corresponding standard deviation (SD) of the 5-fold cross-validation is reported. First ECG approach: the DL models were trained using the first acquired 12-lead ECGs. All ECG approach: the DL models were trained using all acquired 12-lead ECGs (not only the first acquired) per patient. Table S8. Demographics of the study cohort (Leuven [file 12916_2022_2350_MOESM1_ESM.docx]

**Methods**

**Description of normal ECGs by Muse**

General Electronics (GE), the manufacturer of the MUSE ECG system, has developed an extensive algorithm to analyze ECGs made with their machines, called Marquette 12SL [16-17]. This is a top-down algorithm that structurally checks the ECG for a long list of possible abnormalities, ranging from technical inadequacies, arrhythmia, conduction disorders, repolarization changes, ST-elevation/depression among many others. These criteria are well described in the GE Healthcare Marquette™ 12SL™ECG Analysis Program Physician’s Guide [16]. If no abnormalities are to be found, the program labels the ECG as ‘normal’.

**Automatically measured QTc Vs. manually measured QTc**

The automatically measured QT interval from the ECG machine’s algorithm GE Marquette 12SL uses a median beat deriving from three consecutive beats from which the QT interval is measured using the end of the T-wave, also known as the Threshold method. Manual measurements of the QT interval were done using the Tangent method [18]. The QTc was calculated using Bazzet’s formula. Because normal limits vary between these measuring methods, the cut-off for QTc prolongation for the automatically measured QTc is 450 ms for males and 460 ms for females. For the manually measured QTc the cut-off is 440 ms for males and 450 ms for females [19].

**Tables and Figures**

**Table S1. Demographics of the study cohort - Amsterdam data.**

| **Variable** | **LQTS1** | | **LQTS2** | **LQTS3** | **Controls** |
| --- | --- | --- | --- | --- | --- |
| N | | 172 | 214 | 72 | 10000 |
| European (non-Finnish) (n, %) | | 151 (87.8) | 194 (90.7) | 69 (95.8) | NA |
| Greater Middle East (n, %) | | 12 (7.0) | 12 (5.6) | 0 (0.0) | NA |
| East Asian (n, %) | | 4 (2.3) | 3 (1.4) | 1 (1.4) | NA |
| Latino (n, %) | | 1 (0.6) | 1 (0.5) | 0 (0.0) | NA |
| African / African American (n, %) | | 0 (0.0) | 0 | 2 (2.8) | NA |
| Other ethnicity (n, %) | | 4 (2.3) | 4 (1.9) | 0 (0.0) | NA |
| Symptomatic at baseline (n, %) | | 96 (55.8) | 85 (39.7) | 25 (34.7) | NA |
| Symptomatic at baseline or during follow-up (n, %) | | 101 (58.7) | 95 (44.4) | 27 (37.5) | NA |
| Beta blocker use (ever) (n, %) | | 97 (56.4) | 154 (72.0) | 10 (13.9) | NA |
| Pacemaker (n, %) | | 5 (2.9) | 7 (3.3) | 28 (38.9) | NA |
| ICD (n, %) | | 17 (9.9) | 30 (14.0) | 14 (19.4) | NA |
| Age at first ECG (mean±SD) | | 39±17 | 38±17 | 46±18 | 53±17 |
| Sex (n male, %) | | 65 (38) | 89 (41) | 33 (45) | 4300 (43) |
| QTc all (mean±SD)* | | 456±34 | 451±36 | 450±36 | 411±17 |
| QTc female (mean ±SD)* | | 465 ±32 | 453±33 | 448±36 | 413±17 |
| QTc male (mean±SD)* | | 442±32 | 448±40 | 453±36 | 408±16 |
| QTc prolonged (n, %)* | | 87 (51) | 83 (39) | 31 (43) | 133 (0.01) |
| QTc prolonged (n, %)^#^ | | 86 (50) | 107 (50) | 28 (39) | NA |

Symptomatic at baseline means the patient had an arrhythmic even before the diagnosis of LQTS was made, after genetic testing. Symptomatic at baseline or during means the patient either was symptomatic at baseline or became symptomatic after diagnosis was made. ICD = Implantable Cardioverter-Defibrillator. QTc for each ECG was calculated by the ECG machine’s algorithm(1)**.** *The automatically measured QTc was used (thresholds used to define prolonged QTc: ≥ 450 ms for males, QTc ≥ 460 ms for females). ^#^The manually measured QTc was used (thresholds used to define prolonged QTc: ≥ 440 ms for males, QTc ≥ 450 ms for females).

**Table S2.** **Distribution of different types of genetic variants for LQTS 1, 2, and 3 – Amsterdam data.**

| **Data** | **Amsterdam data** | | |
| --- | --- | --- | --- |
| **Variant type** | **LQTS1 (n, %)** | **LQTS2 (n, %)** | **LQTS3 (n, %)** |
| Missense | 146 (84.8) | 131 (61.2) | 42 (58.3) |
| Nonsense | 6 (3.4) | 22 (10.2) | 0 (0.0) |
| Frameshift | 12 (7.0) | 43 (20.6) | 0 (0.0) |
| Deletion | 5 (2.9) | 9 (4.2) | 2 (2.8) |
| Insertion | 1 (0.6) | 0 (0.0) | 27 (37.5) |
| Duplication | 0 (0.0) | 8 (3.7) | 0 (0.0) |
| Combined | 0 (0.0) | 0 (0.0) | 1 (1.4) |
| Unknown | 2 (1.2) | 1 (0.5) | 0 (0.0) |
| Total | 172 | 214 | 72 |

For LQTS3 these are in frame deletions, which is not the case for LQTS1 and 2.

**Table S3:** **Prevalence of specific genetic variants for LQTS 1, 2, and 3 - Amsterdam data.**

| **KCNQ1 (LQTS1)** | | **KCNH2 (LQTS2)** | | **SCN5A (LQTS3)** | |
| --- | --- | --- | --- | --- | --- |
| **variants** | **n (%)** | **variants** | **n (%)** | **variants** | **n (%)** |
| unknown/missing | 2 (1.2) | unknown/missing | 1 ( 0.5) | p.1507_1509delGlnLysPro | 1 ( 1.4) |
| c.1032+5G>A | 7 (4.1) | 3,682 bp duplication | 7 ( 3.3) | p.1507_1509delGlnLysPro and p.IIe1660Val | 1 ( 1.4) |
| c.1515-2_1515-1del | 1 (0.6) | c.2027delAG | 2 ( 0.9) | p.Ala1330Thr | 1 ( 1.4) |
| c.1795-2A>G | 7 (4.1) | c.2399-2A>G | 4 ( 1.9) | p.Arg1635IIe | 1 ( 1.4) |
| c.387-?_1393+?del | 3 (1.7) | c.2959_2960del | 5 ( 2.3) | p.Arg582Cys | 1 ( 1.4) |
| p.Ala341Val | 1 (0.6) | g.150,648,803_150,652,487du | 1 ( 0.5) | p.Asp1114Glu | 6 ( 8.3) |
| p.Ala344Ala GCG>GCC) | 1 (0.6) | p.61n688* | 1 ( 0.5) | p.Gly615Glu | 1 ( 1.4) |
| p.Ala344Val | 16 (9.3) | p.Ala422Asp | 2 ( 0.9) | p.Ile1278Asn | 5 ( 6.9) |
| p.Ala399Ser | 1 (0.6) | p.Ala558Pro | 3 ( 1.4) | p.Ile1768Val | 26 (36.1) |
| p.Arg174His | 2 (1.2) | p.Ala813Cysfs*17 | 3 ( 1.4) | p.Phe1616del | 1 ( 1.4) |
| p.Arg190Gln | 7 (4.1) | p.Arg176Trp | 4 ( 1.9) | p.Tyr1795_Glu1796insAsp | 27 (37.5) |
| p.Arg190Trp | 3 (1.7) | p.Arg242His | 1 ( 0.5) | p.Val456Met | 1 ( 1.4) |
| p.Arg231Cys | 1 (0.6) | p.Arg243Cys | 2 ( 0.9) |  |  |
| p.Arg243Cys | 7 (4.1) | p.Arg252Glyfs*108 | 4 ( 1.9) |  |  |
| p.Arg259Cys | 1 (0.6) | p.Arg328Cys | 3 ( 1.4) |  |  |
| p.Arg366Gln | 3 (1.7) | p.Arg366* | 3 ( 1.4) |  |  |
| p.Arg397Trp | 9 (5.2) | p.Arg534Cys | 6 ( 2.8) |  |  |
| p.Arg562Met | 2 (1.2) | p.Arg582Cys | 27 (12.6) |  |  |
| p.Arg591Cys | 3 (1.7) | p.Arg863* | 2 ( 0.9) |  |  |
| p.Arg594Gln | 3 (1.7) | p.Arg912fs | 4 ( 1.9) |  |  |
| p.Arg595Gln | 4 (2.3) | p.Arg912Glyfs*6 | 1 ( 0.5) |  |  |
| p.Asp564Gly | 1 (0.6) | p.Asp837Asn | 1 ( 0.5) |  |  |
| p.Gln356* | 3 (1.7) | p.Cys566Alafs*28 | 3 ( 1.4) |  |  |
| p.Glu449Argfs*14 | 6 (3.5) | p.Cys64Tyr | 3 ( 1.4) |  |  |
| p.Glu578Val | 1 (0.6) | p.Cys750Arg | 1 ( 0.5) |  |  |
| p.Gly189Arg | 2 (1.2) | p.Glu289* | 4 ( 1.9) |  |  |
| p.Gly189Glu | 6 (3.5) | p.Glu289Lys | 1 ( 0.5) |  |  |
| p.Gly292Asp | 1 (0.6) | p.Glu365* | 1 ( 0.5) |  |  |
| p.Gly314Asp | 1 (0.6) | p.Glu698* | 10 ( 4.7) |  |  |
| p.Gly568Arg | 2 (1.2) | p.Gly1036Alafs*21 | 12 ( 5.6) |  |  |
| p.Ile274Val | 3 (1.7) | p.Gly572Cys | 3 ( 1.4) |  |  |
| p.Lys421* | 2 (1.2) | p.Gly572Ser | 1 ( 0.5) |  |  |
| p.Lys422Thr | 6 (3.5) | p.Gly584Ser | 3 ( 1.4) |  |  |
| p.Phe130Argfs*77 | 6 (3.5) | p.Gly604Ser | 1 ( 0.5) |  |  |
| p.Phe193Leu | 2 (1.2) | p.Gly785Val | 3 ( 1.4) |  |  |
| p.Phe296Ser | 8 (4.7) | p.Gly873Alafs*5 | 9 ( 4.2) |  |  |
| p.Phe34del | 1 (0.6) | p.Gly925Glyfs*49 | 2 ( 0.9) |  |  |
| p.Ser143Phe | 1 (0.6) | p.Gly969Valfs*5 | 1 ( 0.5) |  |  |
| p.Ser225Leu | 1 (0.6) | p.Ile1768Val | 1 ( 0.5) |  |  |
| p.Ser373Pro | 2 (1.2) | p.Ile57Thr | 1 ( 0.5) |  |  |
| p.Ser566Pro | 1 (0.6) | p.Ile642_Val644del | 2 ( 0.9) |  |  |
| p.Thr322Met | 4 (2.3) | p.Ile96Val | 1 ( 0.5) |  |  |
| p.Thr587Met | 1 (0.6) | p.Leu69Pro | 3 ( 1.4) |  |  |
| p.Trp12Cys | 2 (1.2) | p.Leu87Pro | 1 ( 0.5) |  |  |
| p.Trp392Arg | 4 (2.3) | p.Leu97Pro | 2 ( 0.9) |  |  |
| p.Try171* | 1 (0.6) | p.Met645Leu | 1 ( 0.5) |  |  |
| p.Tyr184Ser | 10 (5.8) | p.Phe640Leu | 3 ( 1.4) |  |  |
| p.Tyr315Ser | 1 (0.6) | p.Pro191Argfs*1 | 3 ( 1.4) |  |  |
| p.Val280Ala | 4 (2.3) | p.Pro241Leu | 2 ( 0.9) |  |  |
| p.Val319Leu | 2 (1.2) | p.Pro297Ser | 3 ( 1.4) |  |  |
| p.Val524Gly | 3 (1.7) | p.Pro347Ser | 1 ( 0.5) |  |  |
|  |  | p.Pro72Arg | 1 ( 0.5) |  |  |
|  |  | p.Pro926Alafs*14 | 1 ( 0.5) |  |  |
|  |  | p.Ser283Cysfs*77 | 1 ( 0.5) |  |  |
|  |  | p.Ser602Asn | 2 ( 0.9) |  |  |
|  |  | p.Ser649Leu | 2 ( 0.9) |  |  |
|  |  | p.Ser906Leu | 3 ( 1.4) |  |  |
|  |  | p.Thr613Lys | 3 ( 1.4) |  |  |
|  |  | p.Thr613Met | 2 ( 0.9) |  |  |
|  |  | p.Trp927* | 1 ( 0.5) |  |  |
|  |  | p.Tyr845Asp | 2 ( 0.9) |  |  |
|  |  | p.Tyr99Ser | 24 (11.2) |  |  |
|  |  | p.Val625Glu | 2 ( 0.9) |  |  |
|  |  | p.Val94Ala | 1 ( 0.5) |  |  |

**Table S4. Single Lead First ECG model performances for LQTS1 – Amsterdam data.**

| **Training** | **Validation** | | | |
| --- | --- | --- | --- | --- |
|  | **Sensitivity ± SD** | | **Specificity ± SD** | **AUC ± SD** |
| I (first acquired ECG) | 82±12% | 90±2% | | 0.86±0.06 |
| II (first acquired ECG) | 76±8% | 95±1% | | 0.86±0.04 |
| III (first acquired ECG) | 75±15% | 84±12% | | 0.79±0.07 |
| aVR (first acquired ECG) | 72±15% | 96±1% | | 0.84±0.07 |
| aVL (first acquired ECG) | 70±13% | 91±3% | | 0.81±0.07 |
| aVF (first acquired ECG) | 70±15% | 90±7% | | 0.80±0.05 |
| V1 (first acquired ECG) | 85±4% | 90±5% | | 0.87±0.02 |
| V2 (first acquired ECG) | 73±12% | 96±2% | | 0.85±0.06 |
| V3 (first acquired ECG G) | 73±8% | 92±2% | | 0.81±0.04 |
| V4 (first acquired ECG) | 79±7% | 93±5% | | 0.85±0.03 |
| V5 (first acquired ECG) | 78±8% | 94±1% | | 0.86±4 |
| V6 (first acquired ECG) | 81±9% | 90±7% | | 0.85±0.05 |

The mean of the collected metrics and the corresponding standard deviation (SD) of the 5-fold cross-validation is reported. The DL models were trained using single leads of the first acquired ECG of control and LQTS2 patients.

**Table S5. Single Lead First ECG model performances for LQTS2 – Amsterdam data.**

| **Training** | **Validation** | | |
| --- | --- | --- | --- |
|  | **Sensitivity ± SD** | **Specificity ± SD** | **AUC ± SD** |
| I (first acquired ECG) | 88±3% | 93±3% | 0.91±0.01 |
| II (first acquired ECG) | 87±8% | 91±4% | 0.89±0.04 |
| III (first acquired ECG) | 83±4% | 84±4% | 0.85±0.02 |
| aVR (first acquired ECG) | 89±4% | 93±1% | 0.91±0.02 |
| aVL (first acquired ECG) | 85±10% | 85±7% | 0.85±0.03 |
| aVF (first acquired ECG) | 82±8% | 88±8% | 0.85±0.02 |
| V1 (first acquired ECG) | 87±7% | 89±5% | 0.88±0.01 |
| V2 (first acquired ECG) | 89±6% | 89±6% | 0.89±0.04 |
| V3 (first acquired ECG G) | 88±8% | 87±9% | 0.87±0.03 |
| V4 (first acquired ECG) | 84±7% | 93±4% | 0.88±0.03 |
| V5 (first acquired ECG) | 83±9% | 93±3% | 0.88±0.03 |
| V6 (first acquired ECG) | 84±6% | 88±6% | 0.86±0.03 |

The mean of the collected metrics and the corresponding standard deviation (SD) of the 5-fold cross-validation is reported. The DL models were trained using single leads of the first acquired ECG of control and LQTS2 patients.

**Table S6. Single Lead First ECG model performances for LQTS3 – Amsterdam data.**

| **Training** | **Validation** | | |
| --- | --- | --- | --- |
|  | **Sensitivity ± SD** | **Specificity ± SD** | **AUC ± SD** |
| I (first acquired ECG) | 68±19% | 90±5% | 0.79±0.07 |
| II (first acquired ECG) | 57±22% | 92±3% | 0.75±0.10 |
| III (first acquired ECG) | 62±23% | 80±7% | 0.81±0.09 |
| aVR (first acquired ECG) | 65±12% | 93±4% | 0.79±0.06 |
| aVL (first acquired ECG) | 59±25% | 82±9% | 0.71±0.08 |
| aVF (first acquired ECG) | 48±12% | 91±2% | 0.70±0.06 |
| V1 (first acquired ECG) | 52±21% | 84±18% | 0.68±0.09 |
| V2 (first acquired ECG) | 69±16% | 82±11% | 0.76±0.04 |
| V3 (first acquired ECG G) | 57±17% | 85±12% | 0.71±0.05 |
| V4 (first acquired ECG) | 69±11% | 86±10% | 0.78±0.03 |
| V5 (first acquired ECG) | 65±15% | 87±5% | 0.76±0.06 |
| V6 (first acquired ECG) | 68±11% | 91±6% | 0.79±0.06 |

The mean of the collected metrics and the corresponding standard deviation (SD) of the 5-fold cross-validation is reported. The DL models were trained using single leads of the first acquired ECG of control and LQTS2 patients.

**Table S7. Model performances for LQTS 1, 2, and 3 - Amsterdam data.**

| **Training** | **Type** | **Internal Validation (Amsterdam data)** | | |
| --- | --- | --- | --- | --- |
|  |  | **Sensitivity ± SD** | **Specificity ± SD** | **AUC ± SD** |
| First ECG approach (Amsterdam data) | LQTS1 | 77±11% | 96±1% | 0.86±0.05 |
|  | LQTS2 | 88±6% | 88±4% | 0.88±0.03 |
|  | LQTS3 | 62±24% | 89±9% | 0.77±0.05 |
| All ECG approach (Amsterdam data) | LQTS1 | 77±3% | 95±2% | 0.86±0.03 |
|  | LQTS2 | 90±2% | 94±1% | 0.92±0.01 |
|  | LQTS3 | 83±8% | 90±5% | 0.86±0.03 |

The performance of the DL models for patients whose QTc is within the 10^th^-90^th^ percentile of the overlapping QTc region is reported. The QTc ranges analyzed are 398-475 ms, 403-477 ms, and 403-475 ms for LTS 1, 2, and 3, respectively. The automatically measured QTc was used (thresholds used to define prolonged QTc: ≥ 450 ms for males, QTc ≥ 460 ms for females). The mean of the collected metrics and the corresponding standard deviation (SD) of the 5-fold cross-validation is reported. First ECG approach: the DL models were trained using the first acquired 12-lead ECGs. All ECG approach: the DL models were trained using all acquired 12-lead ECGs (not only the first acquired) per patient.

**Table S8. Demographics of the study cohort - Leuven data.**

| **Variable** | **LQTS1** | | **LQTS2** | **Controls** |
| --- | --- | --- | --- | --- |
| N | 32 | | 80 | 2280 |
| Age (mean±SD) | | 40±18 | 36±16 | 37±17 |
| Sex (n male, %) | | 11 (34) | 43 (54) | 1080 (47) |
| QTc all (mean±SD) | | 481±47 | 464±34 | 419±22 |
| QTc female (mean±SD) | | 478±42 | 471±28 | 424±22 |
| QTc male (mean±SD) | | 487±57 | 458±39 | 415±23 |
| QTc prolonged (n, %) | | 30 (91) | 62 (76) | 148 (0.06) |

The automatically measured QTc was used (thresholds used to define prolonged QTc: ≥ 450 ms for males, QTc ≥ 460 ms for females).

**Table** **S9. Distribution of different types of genetic variants for LQTS 1 and 2 – Leuven data.**

| **Data** | **Leuven data** | |
| --- | --- | --- |
| **Variant type** | **LQTS1 (n, %)** | **LQTS2 (n, %)** |
| Missense | 13 (40.6) | 23 (28.7) |
| Nonsense | 8 (25.0) | 11 (13.7) |
| Frameshift | 8 (25.0) | 39 (48.7) |
| Deletion | 2 (6.2) | 0 (0.0) |
| Insertion | 0 (0.0) | 0 (0.0) |
| Duplication | 0 (0.0) | 0 (0.0) |
| Combined | 1 (3.1) | 7 (8.7) |
| Unknown | 0 (0.0) | 0 (0.0) |
| Total | 32 | 80 |

**Table S10: Prevalence of specific genetic variants for LQTS 1 and 2 - Leuven data.**

| **KCNQ1 (LQTS1)** | | **KCNH2 (LQTS2)** | |
| --- | --- | --- | --- |
| **variants** | **n (%)** | **variants** | **n (%)** |
| c.781-1G>T | 1 ( 3.0) | p.Ala193Glyfs*137 | 14 (17.3) |
| c.921+2T>C | 2 ( 6.1) | p.Ala570Ser | 4 ( 4.9) |
| p.Ala300Thr AND p.Gln376* | 1 ( 3.0) | p.Ala614Val | 1 ( 1.2) |
| p.Ala344Ala (GCG>GCC) | 3 ( 9.1) | p.Arg1014* | 2 ( 2.5) |
| p.Arg231Cys | 1 ( 3.0) | p.Arg582Leu | 7 ( 8.6) |
| p.Arg518* | 1 ( 3.0) | p.Arg863* | 1 ( 1.2) |
| p.Gln356* | 6 (18.2) | p.Asn629Ser | 1 ( 1.2) |
| p.Gln376* | 1 ( 3.0) | p.Asp837His | 3 ( 3.7) |
| p.Ile200Asn | 1 ( 3.0) | p.Cys108Arg | 1 ( 1.2) |
| p.Ile375Argfs*43 | 5 (18.2) | p.Gln884* | 2 ( 2.5) |
| p.Leu496Alafs*19 | 3 ( 9.1) | p.Glu130Lys | 3 ( 3.7) |
| p.Phe339Ser | 1 ( 3.0) | p.Glu807* | 3 ( 3.7) |
| p.Ser225Leu | 1 ( 3.0) | p.Ile512Trpfs*3 | 1 ( 1.2) |
| p.Ser277del | 2 ( 6.1) | p.Leu210dup and p.Ala217Glyfs*121 | 7 ( 8.6) |
| p.Ser566Phe | 1 ( 3.0) | p.Leu987Valfs*131 | 2 ( 2.5) |
| p.Trp305Leu | 1 ( 3.0) | p.Lys21Argfs*5 | 2 ( 2.5) |
| p.Val254Met | 1 ( 3.0) | p.Met651Arg) | 1 ( 1.2) |
|  |  | p.Pro1034Argfs*81 | 1 ( 1.2) |
|  |  | p.Ser621Arg | 1 ( 1.2) |
|  |  | p.Ser818* | 3 ( 3.7) |
|  |  | p.Thr65Pro | 1 ( 1.2) |
|  |  | p.Thr74Cysfs*66 | 8 (11.1) |
|  |  | p.Val680Cysfs*34 | 11 (13.6) |


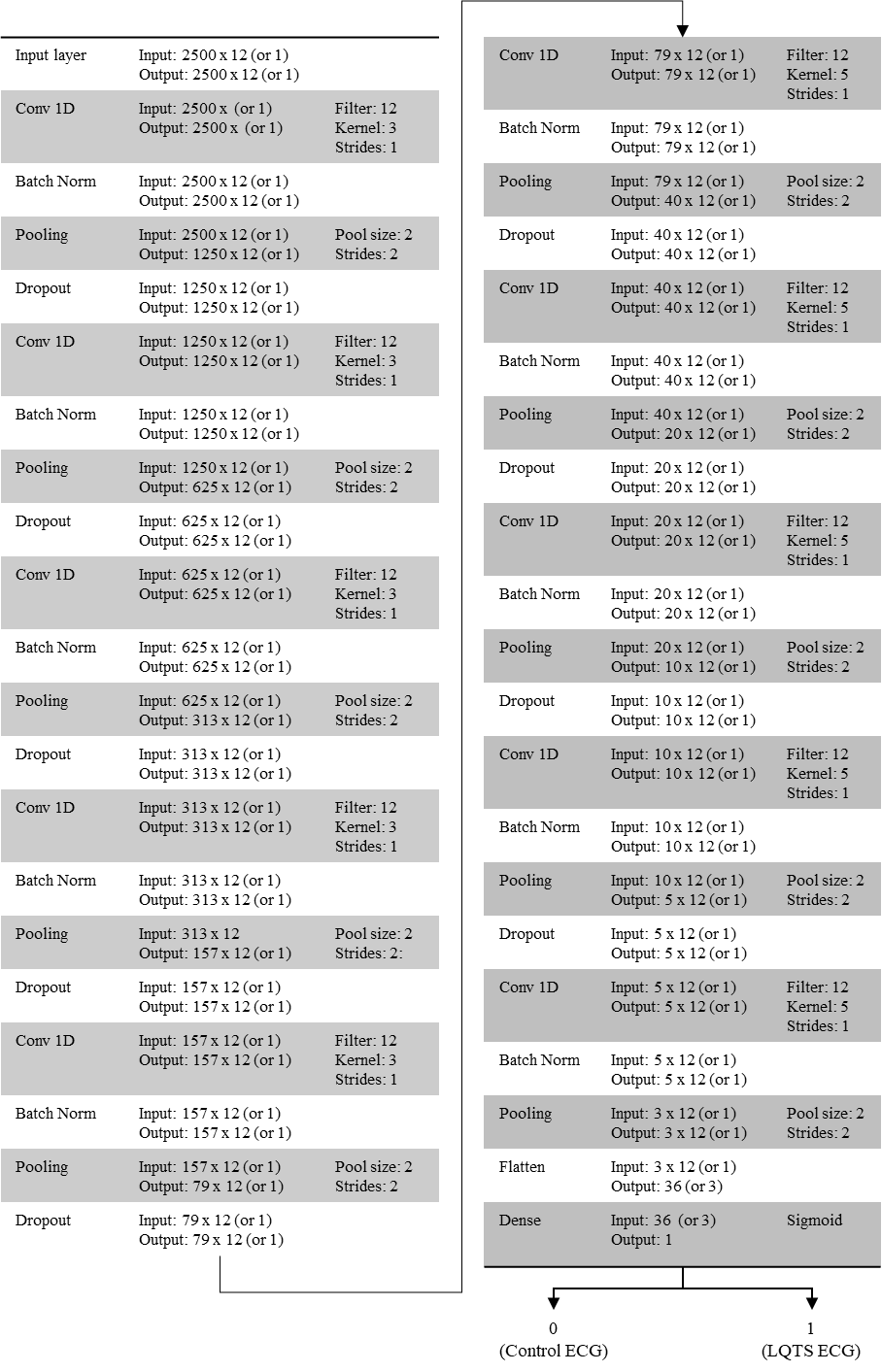


**Fig. S1: 1DCNN** **architecture**. Schematic representation of the proposed 1DCNN architecture. The model was trained using 12-lead ECGs or single lead. The number 12 in the second column represents the 12-lead ECGs, while the number 1 in the bracket represents the use of the single lead. All 12 leads individually were used.

*
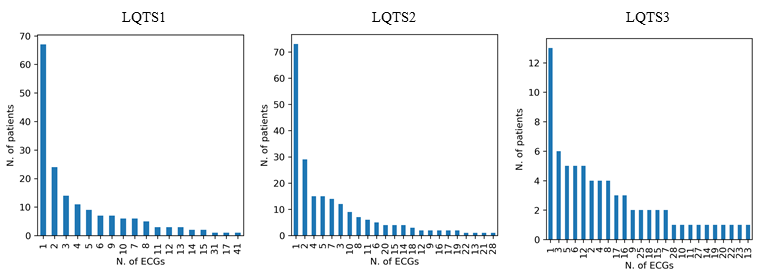
*

**Fig. S2. ECG distribution.** Bar plots showing the number of ECGs for LQTS 1, 2, and 3 patients used in the ALL ECGs approach.


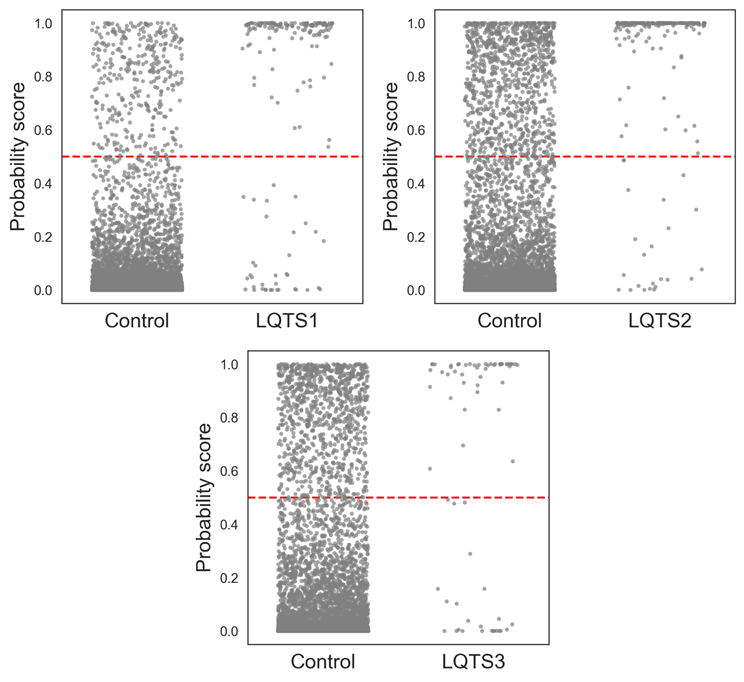


**Fig. S3. Probability score distribution of control and LQTS patients.** Box plots showing the probability score distribution for A) LQTS1, B) LQTS2, and C) LQTS3 and control patients obtained from the DL models trained on the first acquired 12-lead ECGs. The red dotted line represents the threshold used for the ECG classification.


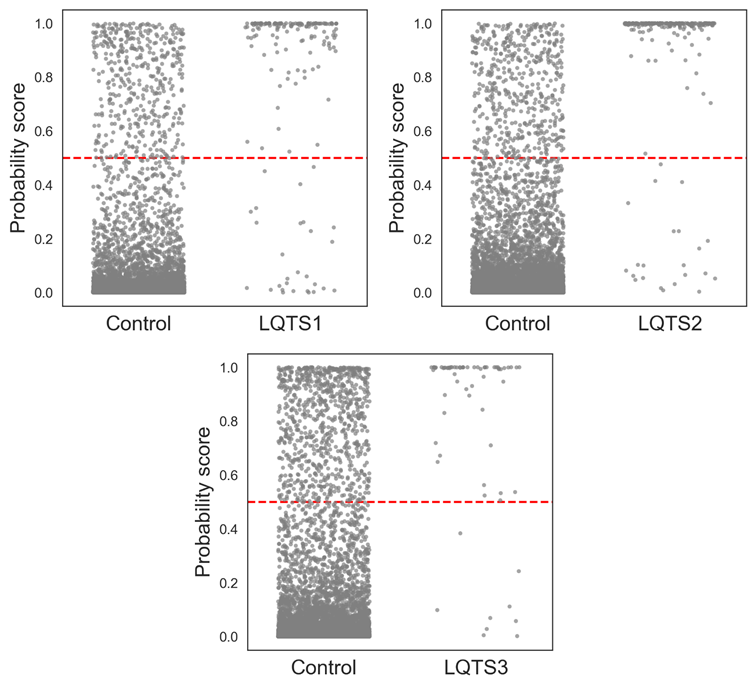


**Fig. S4. Probability score distribution of control and LQTS patients.** Box plots showing the probability score distribution of A) LQTS1, B) LQTS2, and C) LQTS3 and control patients obtained from the DL models trained on all acquired 12-lead ECGs. The red dotted line represents the threshold used for the ECG classification.


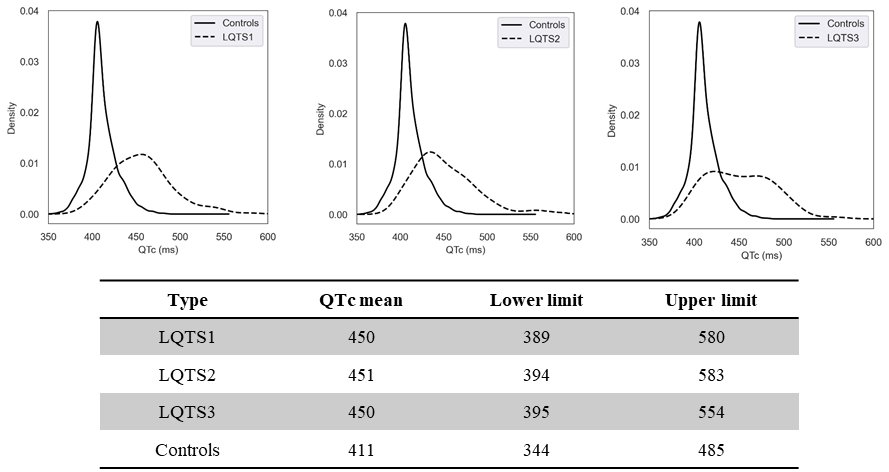


**Fig. S5. QTc value distribution (Amsterdam data).** Top: QTc value distributions of LQTS Vs. control patients. Bottom: table showing the mean, the lower, and the upper limit of the above QTc value distributions. The automatically measured QTc was used (thresholds used to define prolonged QTc: ≥ 450 ms for males, QTc ≥ 460 ms for females).
